# Histone demethylase KDM6B has an anti-tumorigenic function in neuroblastoma by promoting differentiation

**DOI:** 10.1038/s41389-018-0112-0

**Published:** 2019-01-04

**Authors:** Liqun Yang, Yunhong Zha, Jane Ding, Bingwei Ye, Mengling Liu, Chunhong Yan, Zheng Dong, Hongjuan Cui, Han-Fei Ding

**Affiliations:** 1 State Key Laboratory of Silkworm Genome Biology, Southwest University, Chongqing, 400716 China; 2 Institute of Neural Regeneration and Repair and Department of Neurology, The First Hospital of Yichang, Three Gorges University College of Medicine, Yichang, 443000 China; 3 Georgia Cancer Center, Augusta University, Augusta, GA 30912 USA; 4 Department of Biochemistry and Molecular, Medical College of Georgia, Augusta University, Augusta, GA 30912 USA; 5 Department of Cell Biology and Anatomy, Medical College of Georgia, Augusta University, Augusta, GA 30912 USA; 6 Charlie Norwood VA Medical Center, Augusta, GA 30904 USA; 7 Department of Pathology, Medical College of Georgia, Augusta University, Augusta, GA 30912 USA

## Abstract

Induction of differentiation is a therapeutic strategy in high-risk neuroblastoma, a childhood cancer of the sympathetic nervous system. Neuroblastoma differentiation requires transcriptional upregulation of neuronal genes. How this process is regulated at epigenetic levels is not well understood. Here we report that the histone H3 lysine 27 demethylase KDM6B is an epigenetic activator of neuroblastoma cell differentiation. *KDM6B* mRNA expression is downregulated in poorly differentiated high-risk neuroblastomas and upregulated in differentiated tumors, and high *KDM6B* expression is prognostic for better survival in neuroblastoma patients. In neuroblastoma cell lines, KDM6B depletion promotes cell proliferation, whereas KDM6B overexpression induces neuronal differentiation and inhibits cell proliferation and tumorgenicity. Mechanistically, KDM6B epigenetically activates the transcription of neuronal genes by removing the repressive chromatin marker histone H3 lysine 27 trimethylation. In addition, we show that KDM6B functions downstream of the retinoic acid-HOXC9 axis in inducing neuroblastoma cell differentiation: *KDM6B* expression is upregulated by retinoic acid via HOXC9, and KDM6B is required for HOXC9-induced neuroblastoma cell differentiation. Finally, we present evidence that KDM6B interacts with HOXC9 to target neuronal genes for epigenetic activation. These findings identify a KDM6B-dependent epigenetic mechanism in the control of neuroblastoma cell differentiation, providing a rationale for reducing histone H3 lysine 27 trimethylation as a strategy for enhancing differentiation-based therapy in high-risk neuroblastoma.

## Introduction

Neuroblastoma is a common pediatric cancer of the sympathetic nervous system derived from the neural crest cell^1–4^. Approximately half of all neuroblastoma cases are classified as high risk^5^, which have an overall survival rate <50%, even after intensive, multimodal therapy^6,7^. High-risk neuroblastomas are predominantly Schwannian stroma-poor, undifferentiated or poorly differentiated tumors^5,8^, and induction of differentiation by agents, such as *all trans*-retinoic acid (RA)^9,10^, has been exploited as a treatment strategy in high-risk neuroblastoma^11–14^. A better understanding of the molecular basis of neuroblastoma differentiation may suggest new therapeutic targets or strategies for enhancing the efficacy of differentiation-based therapy for high-risk neuroblastoma patients.

Studies of normal stem cell differentiation have demonstrated a pivotal role of epigenetic regulation via histone lysine methylation in the differentiation process^15,16^. For example, trimethylation of histone H3 at lysine 27 (H3K27me3) is required for the transcriptional repression of development genes and the maintenance of pluripotency in embryonic stem cells (ESCs)^17,18^. Interestingly, recent studies have provided evidence for an important role of the H3K27-specific methyltransferase EZH2 in neuroblastoma pathogenesis. It has been reported that high EZH2 expression is prognostic for poor clinical outcome in neuroblastoma patients and that EZH2-catalyzed H3K27me3 is responsible for transcriptional repression of several neuroblastoma tumor-suppressor genes, including *CASZ1 GLU, RUNX3*, and *NGFR*^19^. In addition, Ezh2 activity has been shown to be critical for the growth of mouse neuroblastoma spheres in culture and for tumor development in the *TH-MYCN* mouse model^20^. In line with these findings, a more recent study has revealed a crucial role of EZH2 in blocking neuroblastoma cell differentiation^21^.

Since histone lysine methylation levels are determined by the balance between the activities of histone lysine methyltransferases and demethylases^22,23^, we reasoned that histone lysine demethylases (KDMs) that antagonize the activity of EZH2 by removing H3K27me3 might have an onco-suppressor function in neuroblastoma. The KDM6B family of demethylases KDM6A and KDM6B, also commonly known as UTX and JMJD3, respectively, are responsible for the removal of H3K27me3^23,24^. Our investigation provides evidence in support of this model, revealing an anti-tumorigenic activity of KDM6B in neuroblastoma cells by inducing neuronal differentiation.

## Results

### *KDM6B* expression is downregulated in neuroblastoma stem-like cells and in high-risk neuroblastoma

**Fig. 1:**
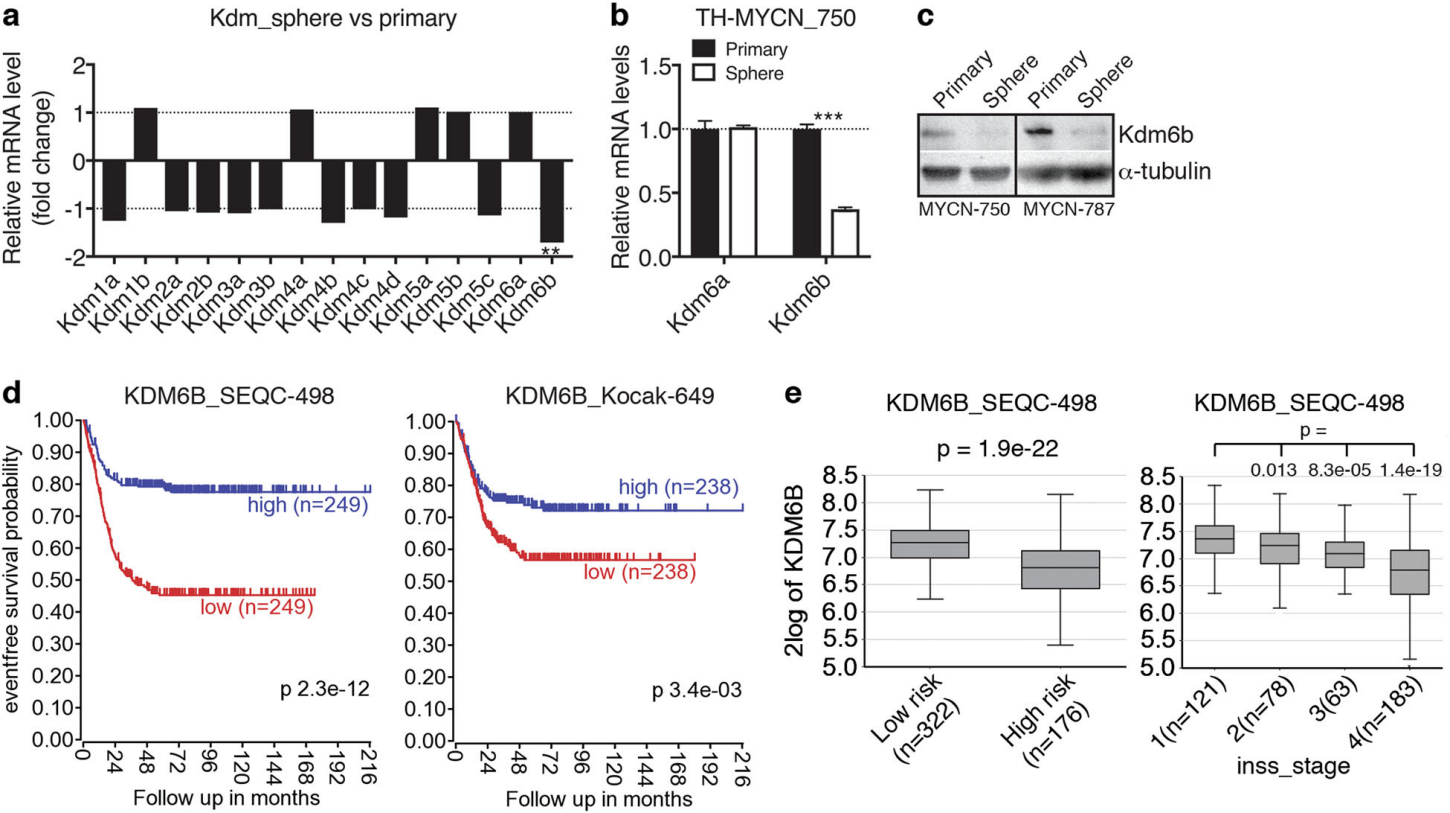
Low *KDM6B* expression is associated with poor prognosis in neuroblastoma patients. **a**–**c** Kdm6b expression is downregulated in mouse neuroblastoma sphere-forming cells as measured by microarray (**a**), qRT-PCR (**b**, error bars, s.d., *n* = 3), and immunoblotting (**c**), in comparison with primary tumor cells. α-tubulin levels are shown as loading control (**c**). Data were analyzed using two-tailed Student’s *t*-test. **d**–**e** Low *KDM6B* expression is associated with reduced event-free survival in neuroblastoma patients (**d**), high-risk neuroblastoma (**e**, left panel), and advanced stages of the tumors (**e**, right panel). Patient data analyses (**d**–**e**) were conducted online (R2 Genomics Analysis and Visualization Platform), and the resulting figures and log-rank test (**d**) and Student’s *t-*test (**e**) *p* values were downloaded. **p < 0.01; ***p < 0.001

We recently reported the isolation and propagation of a population of neuroblastoma sphere-forming cells with cancer stem cell activities, including self-renewal capacity and increased tumorigenic potential, from tumors of the *TH-MYCN* mouse^25^, an animal model of high-risk neuroblastoma with *MYCN* amplification^26–29^. To assess the functions of KDMs in determining neuroblastoma differentiation states, we examined the microarray gene expression profiling data from three independent lines of sphere-forming cells (stem cell state) in comparison with their parental primary tumors (differentiated state)^25^. Strikingly, *Kdm6b* expression was selectively downregulated in sphere-forming cells compared to primary tumor cells, as no significant changes were observed in the expression levels of other demethylase genes examined (Fig. 1a). We verified the observation by quantitative reverse transcriptase-PCR (qRT-PCR) and immunoblot analyses, which showed downregulation of *Kdm6b*, but not *Kdm6a*, mRNA expression (Fig. 1b) and of Kdm6b protein expression (Fig. 1c) in sphere-forming cells relative to their parental primary tumors. Thus, mouse neuroblastoma stem-like cells express significantly lower levels of Kdm6b compared to their more differentiated primary tumor cells.

To assess the clinical relevance of this observation, we examined the correlation between *KDM6B* mRNA expression levels and clinical outcomes using the gene expression profiling data from two independent cohorts of neuroblastoma patients (*n* = 1147)^30,31^. We found that lower *KDM6B* expression is significantly associated with reduced event-free survival of neuroblastoma patients (Fig. 1d), with high-risk neuroblastoma tumors (Fig. 1e, left panel), and with advanced tumor stages (Fig. 1e, right panel). By contrast, *KDM6A* mRNA expression levels showed no correlation with the survival of neuroblastoma patients (Supplementary Fig. 1). Taken together, these results suggest that KDM6B might function as an epigenetic onco-suppressor in the pathogenesis of high-risk neuroblastoma.

### KDM6B inhibits neuroblastoma cell proliferation and tumorigenicity

**Fig. 2:**
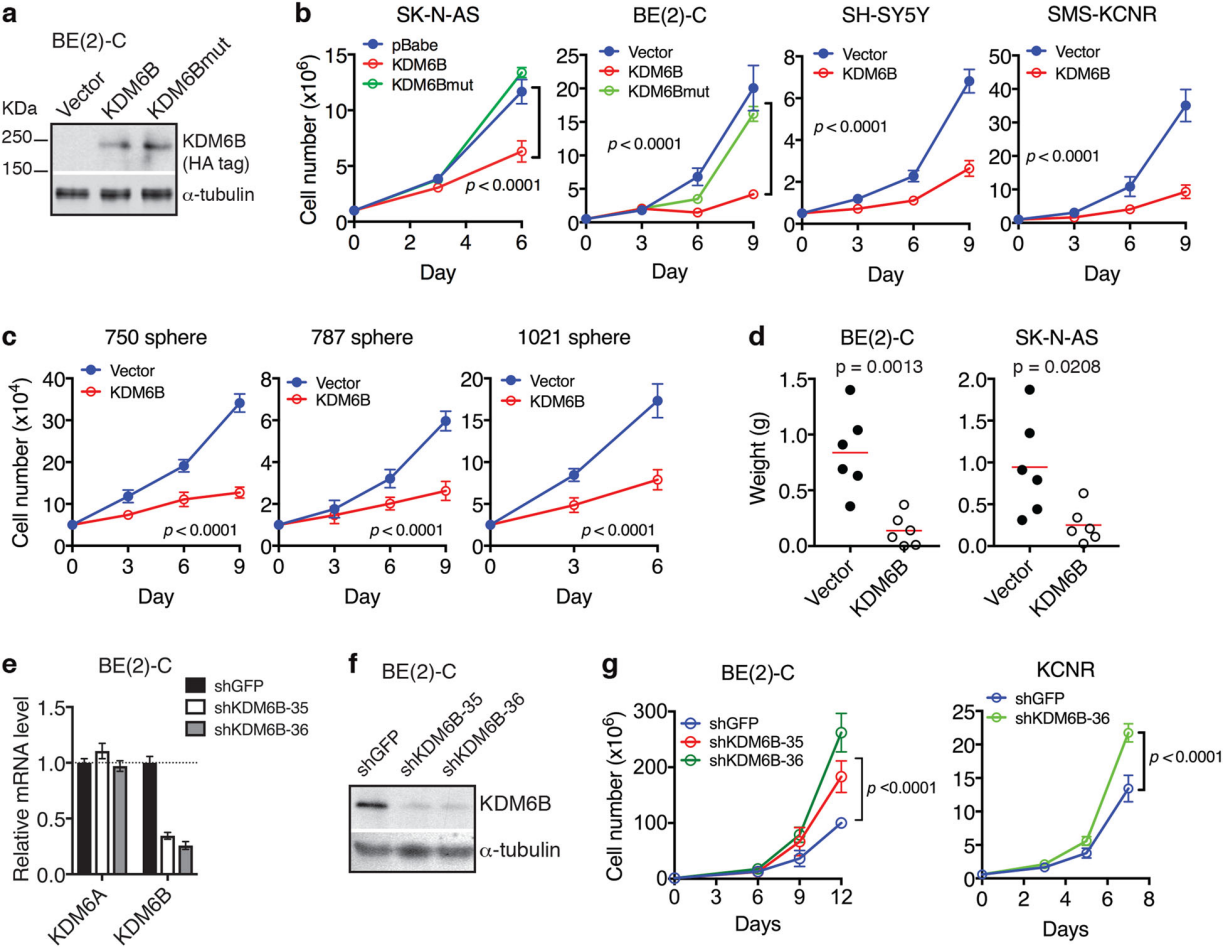
KDM6B inhibits the proliferation and tumorigenicity of neuroblastoma cells. **a** Immunoblot analysis of overexpression of HA-tagged KDM6B in BE(2)-C cells. α-tubulin levels are shown as loading control. **b**–**c** Growth curves of human neuroblastoma cell lines (**b**) and mouse neuroblastoma sphere-forming cell lines (**c**) with or without overexpression of HA-tagged human KDM6B obtained by trypan blue exclusion assay. **d** Xenograft assay of BE(2)-C and SK-N-AS cells without (vector control) or with overexpression of HA-tagged KDM6B. Tumor weight was analyzed by scatter plot with horizontal lines indicating the mean (*n* = 6). Data were analyzed using two-tailed Student’s *t*-test with *p* values indicated. **e**–**f** qRT-PCR (**e**) and immunoblot (**f**) analyses of KDM6B mRNA and protein expression in BE(2)-C cells 7 days after infection with control (shGFP) or shKDM6B-expressing lentiviruses. Values (**e**) correspond to the mean of three technical replicates ± s.d. and are representative of two independent experiments. α-tubulin levels are shown as loading control (**f**). **g** Growth curves of the indicated human neuroblastoma cell lines infected with lentiviruses expressing shGFP (control) or shKDM6B. All cell growth data (**b**, **c**, **g**) correspond to the mean of four technical replicates ± s.d. and are representative of at least two independent experiments. Data were analyzed by two-way ANOVA with *p* values indicated

Given that high *KDM6B* expression is indicative of better prognosis for neuroblastoma patients (Fig. 1d), we investigated the effect of high KDM6B expression on neuroblastoma cell proliferation and tumorigenicity. Ectopic expression of human KDM6B (Fig. 2a) markedly inhibited the proliferation of both *MYCN*-amplified (BE(2)-C and SMS-KCNR) and non-*MYCN*-amplified (SH-SY5Y and SK-N-AS) human neuroblastoma cell lines (Fig. 2b). Overexpression of the demethylase-defective mutant KDM6B-H1390A did not significantly inhibit the growth of SK-N-AS and BE(2)-C cells (Fig. 2a, b, KDM6Bmut), indicating that the demethylase activity of KDM6B is required for its growth-inhibitory effect. Similarly, ectopic expression of human KDM6B in mouse neuroblastoma sphere-forming cells resulted in a marked inhibition of cell growth (Fig. 2c). In line with these results, BE(2)-C and SK-N-AS cells with KDM6B overexpression generated much smaller tumors in immunodeficient mice relative to their vector control counterparts (Fig. 2d), indicating that high KDM6B expression reduced tumorigenicity of neuroblastoma cells.

To assess the effect of endogenous KDM6B expression on neuroblastoma cell proliferation, we used two distinct short hairpin RNA (shRNA) lentiviral constructs to reduce KDM6B expression (Fig. 2e, f), which had no significant effect on KDM6A expression (Fig. 2e). Knockdown of KDM6B expression significantly enhanced the proliferation of BE(2)-C and SMS-KCNR cells (Fig. 2g). Collectively, these results reveal a growth-inhibitory and anti-tumorigenic function of KDM6B in neuroblastoma cells.

### KDM6B promotes neuronal differentiation of neuroblastoma cells

**Fig. 3:**
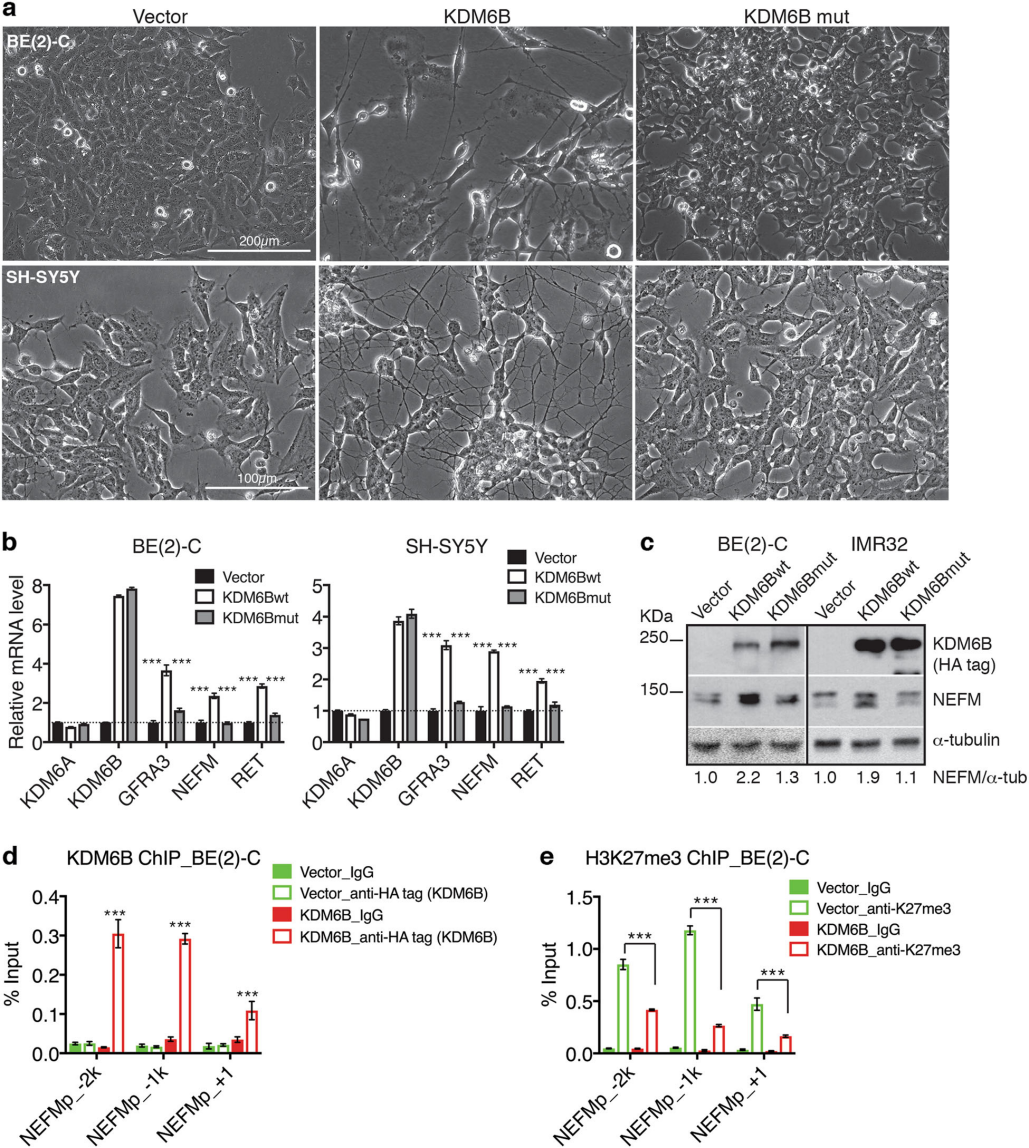
KDM6B induces neuronal differentiation of neuroblastoma cells. **a** Phase contrast images of BE(2)-C and SH-SY5Y cells without (vector control) or with overexpression of HA-tagged KDM6B or KDM6B-H1390A. **b** qRT-PCR analysis of *GFRA3, NEFM*, and *RET* mRNA expression in BE(2)-C and SH-SY5Y cells without (vector control) or with overexpression of HA-tagged KDM6B or KDM6B-H1390A. Error bars represent s.d. (*n* = 3). Data were analyzed by two-tailed Student’s *t*-test. **c** Immunoblot analysis of NEFM levels in BE(2)-C and IMR32 cells without (vector control) or with overexpression of HA-tagged KDM6B or KDM6B-H1390A. NEFM levels were quantified against α-tubulin and are presented as the fraction of the NEFM levels in vector control cells. **d**–**e** ChIP-qPCR analysis of KDM6B (**d**) and H3K27me3 (**e**) levels in the promoter region of *NEFM* in BE(2)-C cells without (vector control) or with overexpression of HA-tagged KDM6B. Data correspond to the mean of three technical replicates±s.d. and are representative of two independent ChIP experiments. Data were analyzed by two-tailed Student’s *t*-test. ***p < 0.001

To gain a molecular understanding of the anti-tumorigenic activity of KDM6B in neuroblastoma cells, we performed gene ontology (GO) analysis of the genes that are co-expressed with *KDM6B* in a cohort of 498 neuroblastoma patients (the SEQC dataset)^30^ (Supplementary Table S1). Our analysis revealed a positive correlation in mRNA expression between *KDM6B* and genes involved in ARF protein signaling, small GTPase signaling, actin cytoskeleton organization, axon genesis, and neuronal differentiation (Supplementary Fig. 2 and Table S2). Of note, the ARF and RAS families of small GTPases have a key role in regulation of cytoskeleton reorganization that is essential for neurogenesis and neurite outgrowth, branching, and retraction^32–35^. Thus, high KDM6B expression is associated with neuroblastoma differentiation. In agreement with the genetic evidence, overexpression of KDM6B in several neuroblastoma cell lines induced neuronal differentiation characterized by extensive neurite outgrowth (Fig. 3a and Supplementary Fig. 3a). We further confirm that these cells underwent neuronal differentiation at the molecular level by qRT-PCR, which showed that overexpression of KDM6B, but not its demethylase-defective mutant, resulted in a significant increase in mRNA levels of neuronal and differentiation marker genes, including neurofilament medium (*NEFM*), glial cell line-derived neurotrophic factor (GDNF) family receptor alpha 3 (*GFRA3*), and *RET* (Fig. 3b). In addition, immunoblot analysis revealed that overexpression of KDM6B, but not its mutant, increased NEFM protein levels in neuroblastoma cells (Fig. 3c). It is known that expression of these genes is increased in neuroblastoma cells undergoing neuronal differentiation induced by RA and HOX proteins^36–40^. NEFM is upregulated during neuronal development and is involved in axon outgrowth and guidance^41^, and GFRA3 forms a receptor complex with RET, which has a critical role in promoting the survival, differentiation, axonal outgrowth, and target innervation of sympathetic neurons^42^.

We next investigated whether KDM6B directly targets neuronal genes, using *NEFM* as a model system. Chromatin immunoprecipitation and quantitative PCR (ChIP-qPCR) revealed that KDM6B overexpression in BE(2)-C cells led to a marked increase in KDM6B levels at the promoter of *NEFM* (Fig. 3d), which was accompanied by a significant reduction in H3K27me3 levels in the promoter region (Fig. 3e). Of note, KDM6B overexpression or downregulation had no significant effect on global H3K27me3 levels (Supplementary Fig. 3b). These observations are consistent with the notion that KDM6B has a key role in the removal of H3K27me3 from lineage genes during differentiation, but has no significant impact on genome-wide H3K27me3 deposition^43^. Collectively, these findings suggest that high levels of KDM6B initiate a neuronal differentiation program in neuroblastoma cells by conferring an epigenetically active chromatin state to neuronal genes for transcriptional activation.

### KDM6B is induced by RA and is a direct transcriptional target of HOXC9

**Fig. 4:**
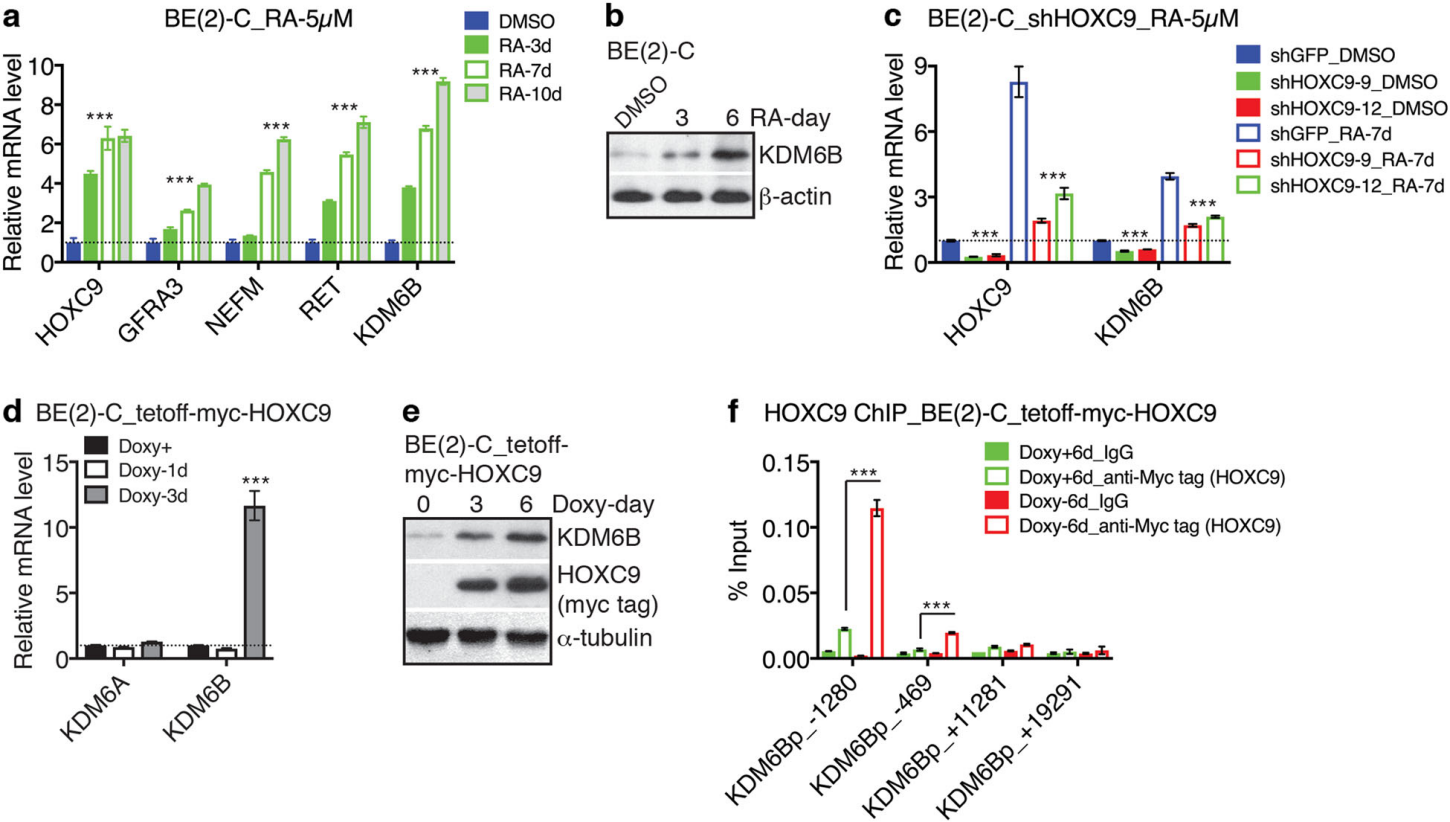
Retinoic acid induces *KDM6B* via HOXC9. **a**–**b** qRT-PCR (**a**) and immunoblot (**b**) analysis of mRNA and protein expression of the indicated genes in BE(2)-C cells treated with vehicle (DMSO) or 5 µM RA for the indicted times. β-actin levels are shown as loading control (**b**). **c** qRT-PCR analysis of *HOXC9* and *KDM6B* mRNA expression in BE(2)-C cells infected with lentiviruses expressing shGFP or shHOXC9 and treated with DMSO or 5 µM RA for 7 days. **d**–**e** qRT-PCR analysis of *KDM6A* and *KDM6B* mRNA expression (**d**) and immunoblot analysis of KDM6B and HOXC9 protein expression (**e**) in BE(2)-C cells with inducible HOXC9 expression in the absence of doxycycline (Doxy). All qRT-PCR data are presented as mean ± s.d. (*n* = 3). α-tubulin levels are shown as loading control (**e**). **f** ChIP-qPCR analysis of HOXC9 levels at the *KDM6B* locus in BE(2)-C cells with inducible expression of Myc-tagged HOXC9 in the absence of Doxy. Data correspond to the mean of three technical replicates±s.d. and are representative of two independent ChIP experiments. All data were analyzed by two-tailed Student’s *t*-test. ***p < 0.001

The above observation that KDM6B overexpression was sufficient to induce neuroblastoma cell differentiation prompted us to investigate whether KDM6B has a role in the action of RA, which is commonly used for induction of neuroblastoma cell differentiation^9,10^ and has been used in clinic for treatment of high-risk neuroblastomas^11–14^. We have shown previously that HOXC9 is a major mediator of RA action in inducing neuroblastoma cell differentiation^38–40^. As expected, RA treatment resulted in upregulation of HOXC9 and neuronal genes, including *GFRA3*, *NEFM*, and *RET* (Fig. 4a). We found that RA treatment also increased KDM6B mRNA and protein expression in a time-dependent manner (Fig. 4a, b). Importantly, RA induction of *KDM6B* mRNA was dependent on HOXC9 since knockdown of HOXC9 expression by shRNA significantly reduced the levels of *KDM6B* induction (Fig. 4c).

We noticed that HOXC9 knockdown alone led to a significant decrease in *KDM6B* mRNA levels in the absence of RA treatment (Fig. 4c), indicating an essential role of HOXC9 in maintaining the steady-state level of *KDM6B* mRNA expression in neuroblastoma cells. Moreover, HOXC9 overexpression was sufficient to induce KDM6B mRNA and protein expression (Fig. 4d, e), but had no effect on *KDM6A* mRNA expression (Fig. 4d). In agreement with these experimental data, we found that higher *HOXC9* mRNA expression is significantly associated with higher *KDM6B* mRNA expression in primary neuroblastoma tumors (Supplementary Figure 4). By contrast, there is no significant correlation in *HOXC9* and *KDM6A* expression in these tumors (Supplementary Figure 4).

HOX proteins bind DNA sequences with a consensus TAATT/AA-motif^44,45^. In BE(2)-C cells with HOXC9 overexpression, ChIP-qPCR analysis detected significant levels of HOXC9 associated with the promoter of *KDM6B*, particularly around the potential HOX-binding sequence TAATTG starting at 1232 bases upstream of the transcription start site (+1) (Fig. 4f). We found no significant levels of HOXC9 enrichment in other regions of the *KDM6B* locus examined, including exon 5 that encodes the first 46 amino acids (Fig. 4f, KDM6Bp_+11281) and the 3′ untranslated region (Fig. 4f, KDM6Bp_+19291). Thus, HOXC9 binding to the promoter region of *KDM6B* is highly specific. Taken together, these data provide evidence for *KDM6B* as a direct transcriptional target gene of HOXC9 in RA-induced neuronal differentiation of neuroblastoma cells.

### KDM6B is required for HOXC9-induced neuronal differentiation of neuroblastoma cells

**Fig. 5:**
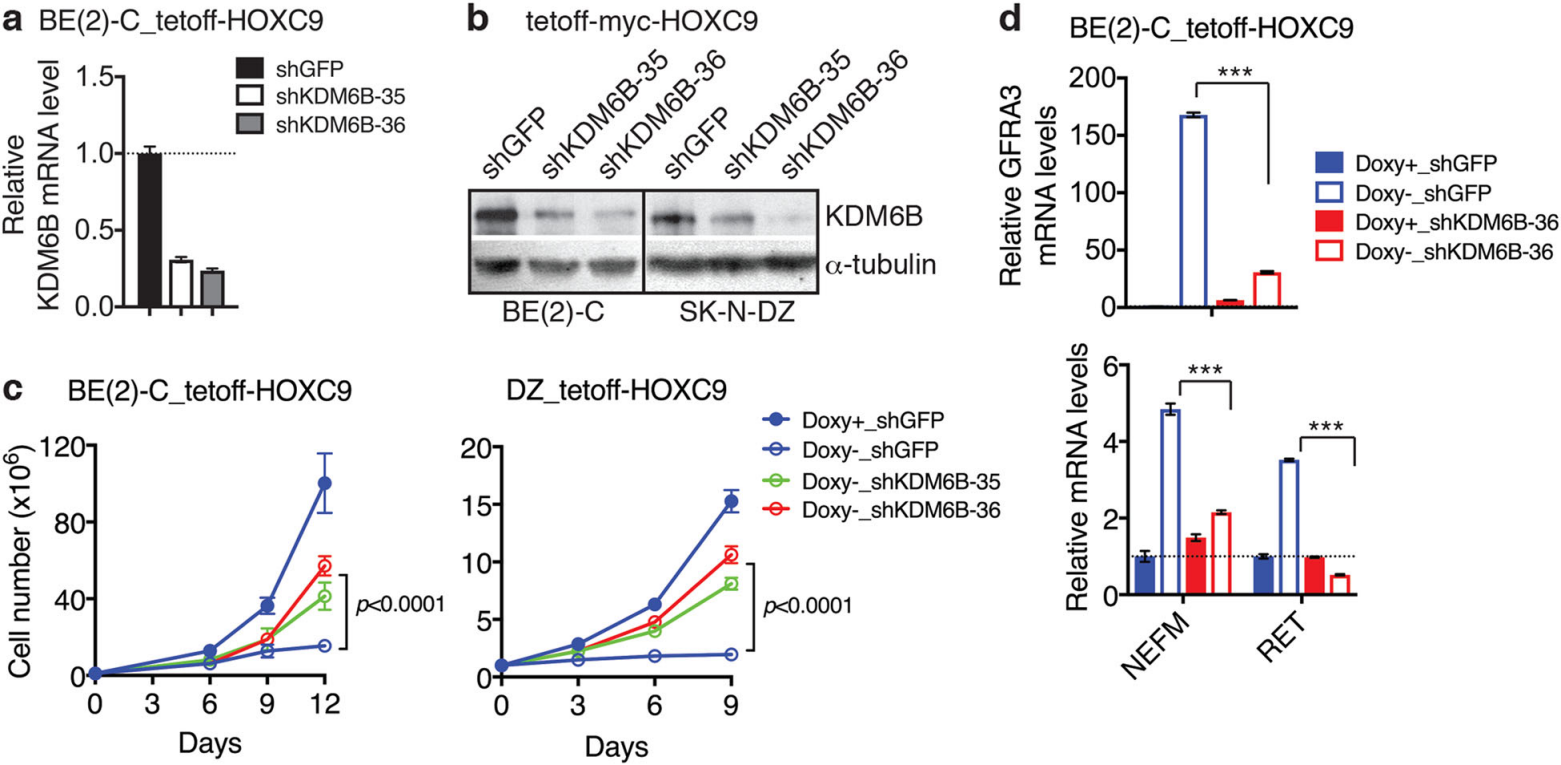
KDM6B is required for HOXC9-induced neuronal differentiation. **a**–**b** qRT-PCR (**a**) and immunoblot (**b**) analyses of KDM6B mRNA and protein expression in BE(2)-C_ and SK-N-DZ_tetoff-myc-HOXC9 cells infected with lentiviruses expressing shGFP or shKDM6B. Error bars represent s.d. (*n* = 3). α-tubulin levels are shown as loading control (**b**). **c** Growth curves of BE(2)-C_ and SK-N-DZ_tetoff-myc-HOXC9 cells expressing either shGFP or shKDM6B-36 that were cultured in the presence or absence of Doxy for the indicted times. Data correspond to the mean of four technical replicates ± s.d. and are representative of at least two independent experiments. Data were analyzed by two-way ANOVA with *p* values indicated. **d** qRT-PCR analysis of *GFRA3, NEFM*, and *RET* mRNA expression in BE(2)-C_tetoff-myc-HOXC9 cells expressing either shGFP or shKDM6B-36 that were cultured in the presence or absence of doxycycline (Doxy) for 9 days. Data were analyzed by two-tailed Student’s *t*-test. ***p < 0.001

HOXC9 is induced by RA and is a major downstream mediator of RA action, and HOXC9 overexpression is sufficient to induce neuronal differentiation of neuroblastoma cells^38,39^. Since HOXC9 activates *KDM6B* transcription, we asked whether KDM6B upregulation is required for HOXC9-induced differentiation. We used shRNA lentiviral constructs to reduce KDM6B mRNA and protein expression in neuroblastoma cell lines with inducible HOXC9 expression (Fig. 5a, b). As expected^38,39^, BE(2)-C_tetoff-myc-HOXC9 cells cultured in the absence of doxycycline underwent neuronal differentiation, showing growth arrest and extensive neurite outgrowth (Fig. 5c and Supplementary Fig. 5). Knockdown of KDM6B expression blocked HOXC9-induced differentiation, as evidenced by the lack of growth arrest and neurite outgrowth of shKDM6B-expressing cells following HOXC9 induction (Fig. 5c and Supplementary Fig. 5). We obtained similar results with SK-N-DZ_tetoff-myc-HOXC9 cells (Fig. 5c). At the molecular level, KDM6B knockdown largely abolished the ability of HOXC9 to upregulate neuronal genes, including *GFRA3, NEFM*, and *RET* (Fig. 5d). These findings indicate that KDM6B is a key mediator of HOXC9 action in induction of neuroblastoma cell differentiation.

### KDM6B interacts with and requires HOXC9 to target neuronal genes for epigenetic activation

**Fig. 6:**
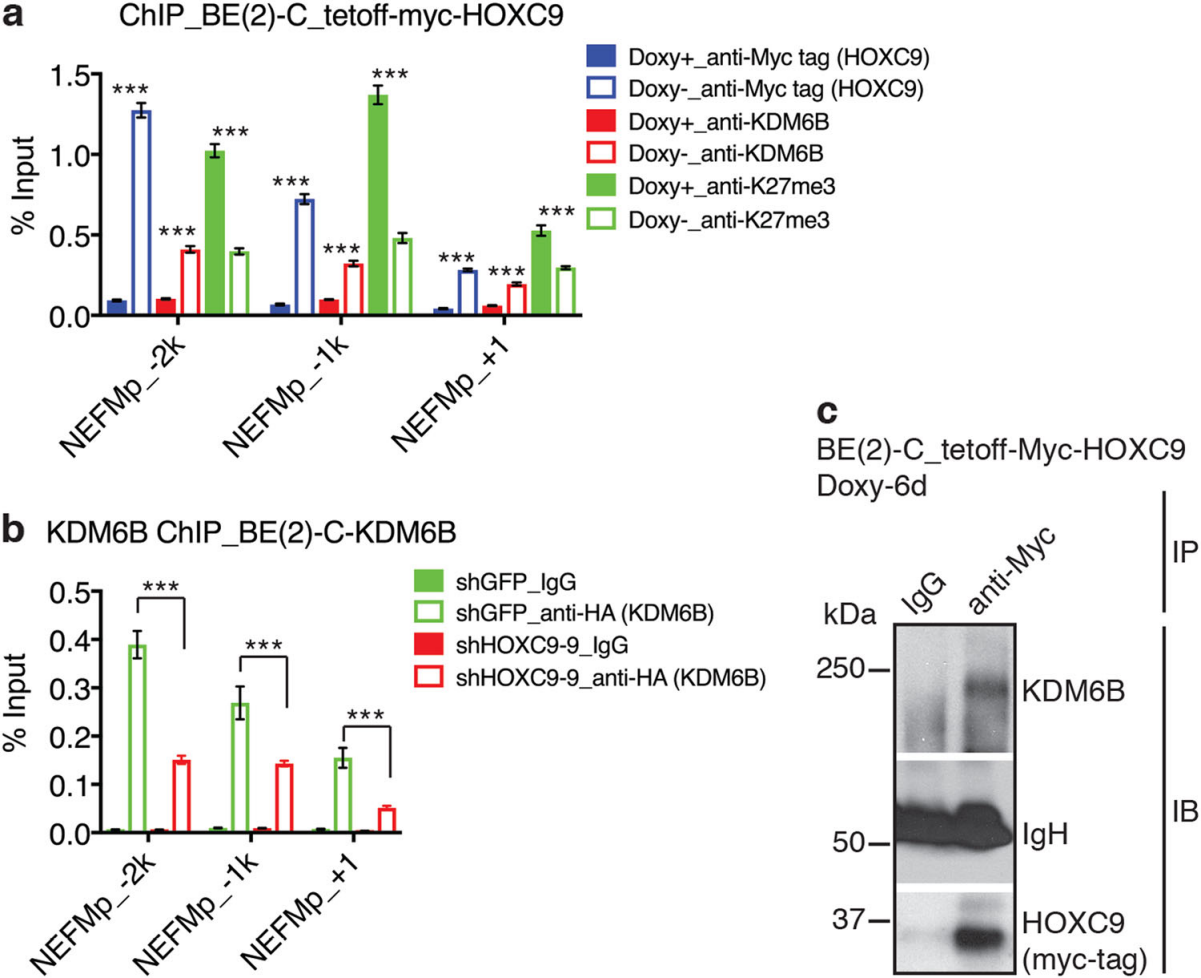
KDM6B interacts with HOXC9 for targeting neuronal genes for epigenetic activation. **a** ChIP-qPCR analysis of HOXC9, KDM6B, and H3K27me3 levels in the promoter region of *NEFM* in BE(2)-C_tetoff-myc-HOXC9 cells that were cultured in the presence of absence of Doxy for 6 days. **b** ChIP-qPCR analysis of KDM6B levels in the promoter region of *NEFM* in BE(2)-C cells with overexpression of HA-tagged KDM6B without (shGFP) or with HOXC9 knockdown by shHOXC9-9. All ChIP-qPCR data correspond to the mean of three technical replicates ±s.d. and are representative of two independent ChIP experiments. Data were analyzed by two-tailed Student’s *t*-test. ***p < 0.001. **c** Co-IP showing HOXC9 interaction with endogenous KDM6B in BE(2)-C_tetoff-myc-HOXC9 cells following HOXC9 induction in the absence of Doxy for 6 days. IgH immunoglobulin heavy chain

To gain further understanding of the molecular basis of KDM6B action in promoting differentiation, we investigated how KDM6B is targeted to neuronal genes during HOXC9-induced differentiation, again using the *NEFM* promoter as a model. We have shown previously that HOXC9 binds to the consensus TAATTA sequence located ~1.9-kb upstream of the *NEFM* gene transcription start site^38^. As expected, HOXC9 overexpression led to a marked increase in HOXC9 levels at the *NEFM* promoter (Fig. 6a, blue columns). Importantly, the levels of endogenous KDM6B at the *NEFM* promoter were also significantly increased (Fig. 6a, red columns), accompanied by a marked reduction in H3K27me3 levels (Fig. 6a, green columns). Thus, HOXC9 overexpression increases KDM6B binding to the *NEFM* promoter.

**Fig. 7:**
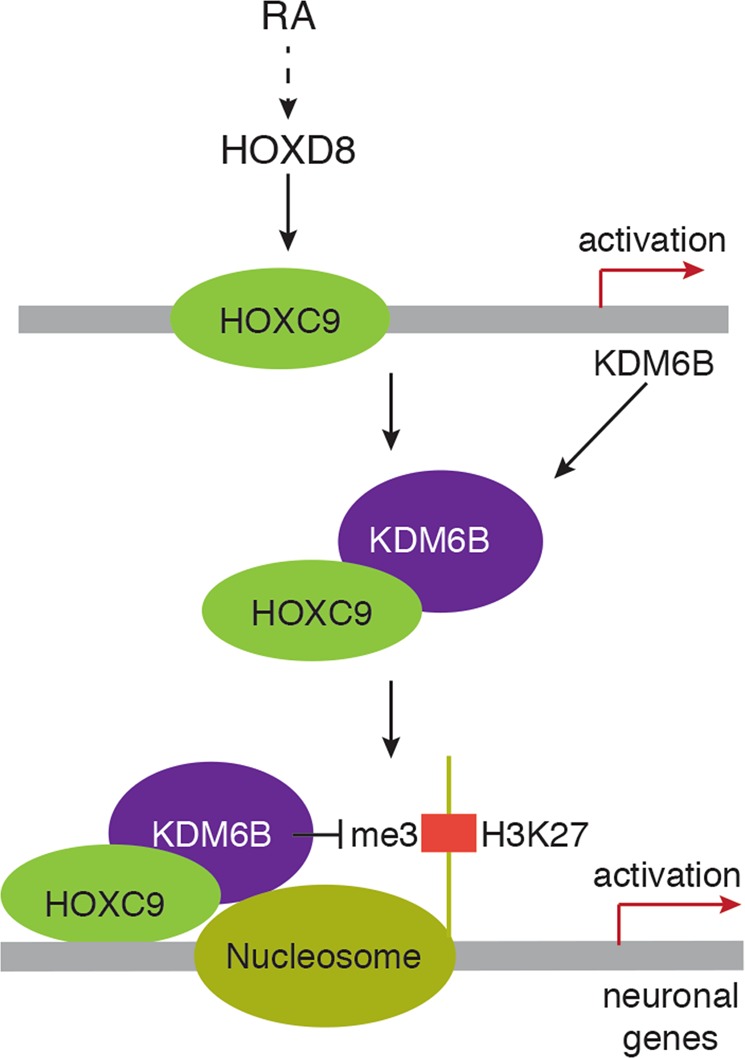
Model for HOXC9-KDM6B cooperation in RA induction of neuronal differentiation in neuroblastoma cells. RA induces HOXD8, which, in turn, transcriptionally activates HOXC9 expression. HOXC9 then activates *KDM6B* transcription and interacts with KDM6B for targeting neuronal genes for epigenetic activation by removing the repressive chromatin marker H3K27me3

In parallel studies, we used shRNA lentiviral constructs to downregulate HOXC9 in BE(2)-C cells overexpressing KDM6B. As shown above (Fig. 3d), KDM6B overexpression resulted in a significant increase in KDM6B levels at the *NEFM* promoter (Fig. 6b). This increase was significantly abolished by knockdown of HOXC9 expression (Fig. 6b). Moreover, we found that endogenous KDM6B could be co-immunoprecipitated with HOXC9 under the condition of HOXC9 overexpression (Fig. 6c). Together, these results suggest that HOXC9 interacts with and recruits KDM6B to neuronal gene promoters for epigenetic activation of gene expression (Fig. 7).

## Discussion

In this report, we present evidence for KDM6B being an epigenetic activator of neuroblastoma cell differentiation. We show that *KDM6B* is downregulated in neuroblastoma stem-like cells and in poorly differentiated, high-risk neuroblastoma tumors. Moreover, higher *KDM6B* expression is correlated with higher expression of genes that promote neuronal differentiation and is prognostic for better survival in neuroblastoma patients. Finally, in a panel of neuroblastoma cell lines, we show that KDM6B depletion enhanced cell proliferation, whereas KDM6B overexpression induced neuronal differentiation and inhibited the proliferation and tumorigenicity of neuroblastoma cells. Collectively, these findings suggest an onco-suppressor function of KDM6B in neuroblastoma pathogenesis by promoting differentiation. Our findings, together with those from recent studies on EZH2^19–21^, indicate that modulation of the H3K27 methylation status is a key epigenetic event in neuroblastoma development.

Induction of differentiation by RA is a therapeutic strategy against high-risk neuroblastoma^11,12^, which, in combination with bone marrow transplant, can significantly improve the event-free survival of high-risk neuroblastoma patients^13,14^. However, resistance to RA presents a major barrier to successful RA-based therapy^11,12^. Identification of downstream mediators of RA action may suggest new strategies to bypass resistance to RA or to enhance its therapeutic effect. We recently identified an RA-HOXD8-HOXC9 axis in driving neuroblastoma cell differentiation:^38–40^ RA induces HOXD8, which, in turn, transcriptionally activates HOXC9 expression. Knockdown of either HOXD8 or HOXC9 expression confers resistance to RA-induced differentiation and forced expression of HOXD8 or HOXC9 can recapitulate the differentiation phenotype induced by RA. Data from the current study reveal an additional step in this molecular pathway, identifying KDM6B as a component of the axis downstream of HOXC9 (Fig. 7). RA induces KDM6B in a HOXC9-dependent manner, and HOXC9 binds to the *KDM6B* promoter to transcriptionally activate *KDM6B* expression. In turn, KDM6B epigenetically activates a neuronal differentiation program by removing the repressive marker H3K27me3 from neuronal genes. Moreover, our data suggest that HOXC9 and KDM6B cooperate in driving this differentiation program: HOXC9 upregulates and requires KDM6B to induce neuronal genes, and KDM6B requires HOXC9 for targeting neuronal genes for epigenetic activation.

The KDM6 family consists of two members, KDM6A and KDM6B. Although both can remove H3K27me3, our data suggest that KDM6A is unlikely to have a significant role in regulation of neuroblastoma cell differentiation. *KDM6A* expression was not correlated with the differentiation state of neuroblastoma tumors and was not upregulated during neuronal differentiation of neuroblastoma cells induced by RA or HOXC9. Also, KDM6B knockdown did not induce KDM6A in neuroblastoma cells. Our observations are consistent with experimental evidence suggesting that KDM6A and KDM6B may function in different developmental lineages. For example, KDM6A is essential for cardiac differentiation of ESCs and for heart development in vivo, but is dispensable for neuronal differentiation of ESCs^46^. By contrast, KDM6B is required for neuronal differentiation of ESCs and neurogenesis in the brain^47,48^. This lineage-dependent activity of KDM6B might also help explain why KDM6B is oncogenic in some cancers, such as T-cell acute lymphocytic leukemia^49^, but anti-oncogenic in neuroblastoma. This notion suggests that targeting KDM6B might offer a strategy to selectively activate differentiation of neuroblastoma cells.

In summary, our study provides clinical and experimental evidence for KDM6B as an epigenetic onco-suppressor in high-risk neuroblastoma by activating a neuronal differentiation program. The findings that either inhibition of EZH2^21^ or activation of KDM6B, as reported here, was sufficient to induce neuroblastoma differentiation suggest that reducing H3K27me3 levels could be a differentiation-based therapeutic strategy in high-risk neuroblastoma, particularly in combination with RA or other differentiation-inducing agents.

## Materials and methods

### Cell lines and culture

Neuroblastoma cell lines BE(2)-C (CRL-2268), IMR32 (CCL-127), SH-SY5Y (CRL-2266), SK-N-AS (CRL-2137), and SK-N-DZ (CRL-2149) were obtained from ATCC (Manassas, VA), LA1-55n from Sigma-Aldrich (06041203, St. Louis, MO), and SMS-KCNR from the Childhood Cancer Repository at Texas Tech University Health Sciences Center. IMR32, SH-SY5Y, SK-N-AS, and SK-N-DZ were cultured in DMEM (HyClone SH30022); BE(2)-C in DME/F-12 1:1 (HyClone SH30023); and LA1-55n and SMS-KCNR in RPMI 1640 (HyClone SH30027) from Thermo Fisher Scientific (Waltham, MA). All media were supplemented with 10% FBS (S11050, Atlanta Biologicals, Flowery Branch, GA). Mouse neuroblastoma sphere-forming cell lines were established as described previously^25^ and were cultured in complete neural crest cell culture medium^50^. For cell growth analysis, numbers of viable cells were determined by trypan blue exclusion assay. For differentiation assay, cells were treated with 5 µM *all trans*-retinoic acid (R2625, Sigma-Aldrich), with 0.1% DMSO as vehicle control. Phase contrast images were captured using an EVOS digital inverted microscope (Advanced Microscopy Group, Mill Creek, WA) or an Axio Observer microscope and AxioVision software (Carl Zeiss MicroImaging, Thornwood, NY).

### Overexpression and RNA interference

Retroviral constructs for expressing human KDM6B (MSCV-JMJD3, Addgene #21212) and its demethylase-defective mutant KDM6B-H1390A (MSCV-JMJD3 mutant, Addgene #21214)^51^ were obtained from Addgene (Cambridge, MA) and verified by DNA sequencing. Lentiviral shRNA constructs targeting human KDM6B (SHCLNG-NM_001080424; shKDM6B-35, TRCN0000359975, targeting sequence, AGATTCTTTCTATGGGCTTTA; shKDM6B-36, TRCN0000367906, targeting sequence, GGAGACCTCGTGTGGATTAAT) were obtained from Sigma-Aldrich. Lentiviral constructs expressing shRNA to HOXC9 have been described previously^38^. BE(2)-C and SK-N-DZ cell lines with inducible expression of Myc-tagged HOXC9 in the absence of doxycycline (Doxy) were established as previously described^38^. Retroviruses were produced in 293FT cells using the packaging plasmids pHDM-G and pMD.MLVogp, and lentiviruses were produced in 293FT cells using the packaging plasmids pLP1, pLP2, and pLP/VSVG (Thermo Fisher Scientific).

### Immunoblotting

Proteins (20–40 µg) were separated on SDS-polyacrylamide gels, transferred to nitrocellulose membranes, and probed with rabbit anti-HA tag (1:1000, C29F4, Cell Signaling, Danvers, MA), rabbit anti-KDM6B (1:1000, GTX124222, GeneTex, Irvine, CA), mouse anti-NEFM (1:200, NF-09, sc-51683, Santa Cruz Biotechnology, Dallas, TX) or mouse anti-α-tubulin (1:5000, B-5-1-2, Sigma-Aldrich). Histones were extracted using the EpiQuik total histone extraction kit (EpiGentek, Farmingdale, NY) and analyzed by immunoblotting using mouse anti-histone H3 (1:1000, 05-499, Millipore, Burlington, MA), rabbit anti-H3K27me3 (1:1000, 07-449, Millipore) or rabbit anti-H3K4me3 (1:1000, ab8895, Abcam, Cambridge, MA). Horseradish peroxidase-conjugated goat anti-mouse and anti-rabbit IgG (Santa Cruz Biotechnology) were used as secondary antibodies. Proteins were visualized using a Clarity Western ECL kit (#1705061, Bio-RAD, Hercules, CA). For visualization using the Odyssey system (LI-COR, Lincoln, NE), goat anti-mouse IRDye 800 or 680 and anti-rabbit IRDye 800 or 680 from LI-COR were used as secondary antibodies.

### qRT-PCR

Total RNA was isolated from cells using Trizol (Invitrogen, Carlsbad, CA), and reverse transcription was performed using SuperScript II Reverse Transcriptase (Invitrogen). qRT-PCR was performed using a RT^2^ SYBR green/Fluorescein PCR master mix (Qiagen, Germantown, MD) on an iQ5 real-time PCR system (Bio-Rad) with primers against various genes (Table S3). All primer pairs were verified by melting curve analysis following qRT-PCR, with each primer pair showing a single desired amplification peak. All samples were normalized to β2 microglobulin (B2M) mRNA levels.

### ChIP-qPCR

ChIP was performed as described^39,52^. Cross-linked chromatin was sheared by sonication and immunoprecipitated using ChIP grade rabbit anti-HA tag (C29F4, Cell Signaling), rabbit anti-histone H3 (ab1791, Abcam), rabbit anti-H3K27me3 (07-449, Millipore), mouse anti-Myc tag (clone 4A6, Millipore) or control mouse or rabbit IgG (Santa Cruz Biotechnology). For qPCR, two independent ChIP samples were analyzed, and each sample was assayed in triplicate using primers that cover the promoter, coding and/or 3′ untranslated regions of *KDM6B* and *NEFM* (Table S4). Data were presented as percentage of the input chromatin (bound/input × 100). For ChIP against H3K27me3, data were normalized to histone H3 content obtained by anti-histone H3 ChIP.

### Co-immunoprecipitation (Co-IP)

Co-IP was performed with a Dynabeads Co-IP kit (10007D, Thermo Fisher Scientific) using the detergent lysis method with extraction buffer containing 150 mM NaCl and 10 µg/ml DNase I. BE(2)-C_tetoff-myc-HOXC9 cells were cultured in the absence of doxycycline for 6 days. Extracts from approximately 1 × 10^7^ cells were incubated overnight at 4 °C with Protein G Dynabeads coated with 2 µg of mouse anti-Myc tag (clone 4A6, Millipore) or mouse IgG (sc-2025, Santa Cruz Biotechnology). After washing with extraction buffer, the beads were suspended in standard SDS sample buffer and analyzed by immunoblotting using rabbit anti-KDM6B (GTX124222, GeneTex), mouse anti-Myc tag or Horseradish peroxidase-conjugated goat anti-mouse IgG (sc-2005, Santa Cruz Biotechnology).

### Xenograft

Approximately 5 × 10^6^ neuroblastoma cells in 200 µl serum-free DMEM were injected subcutaneously into flanks of 6-week-old female NOD.SCID/NCr mice (NOD.CB17-Prkdc^scid^/J, stock number 001303, Jackson Laboratory, Bar Harbor, ME). Approximately 4 weeks after injection, tumors were removed and weighed. All animal experiments were pre-approved by the Institutional Animal Care and Use Committee of Medical College of Georgia, Augusta University.

### Patient data

Patient data used in this study were described previously^30,31^. All analyses were conducted online using R2: Genomics Analysis and Visualization Platform (http://r2.amc.nl), and the resulting figures and *p* value were downloaded.

### Statistics

Quantitative data are presented as mean ± s.d. Cell growth, qRT-PCR, and ChIP-qPCR results are representative of at least two independent experiments with each experiment being conducted in three or four technical replicates (*n* values in the corresponding figure legends). All quantitative data showed apparent normal distribution and equal variance. Statistical significance was estimated using Student’s *t*-test (two-tailed, unpaired) for qRT-PCR and ChIP-qPCR data, and two-way ANOVA for cell growth data. Statistical analyses were conducted using Prism v7.0d for Mac (GraphPad Software, La Jolla, CA).

## Supplementary information

Supplementary Materials

Supplementary Table 1

Supplementary Table 2
