# Supplementary material for: Histone demethylase KDM6B has an anti-tumorigenic function in neuroblastoma by promoting differentiation
Source: Oncogenesis. 2019 Jan 4;8(1):3. doi: 10.1038/s41389-018-0112-0 (PMC6328563; doi:10.1038/s41389-018-0112-0)
Supplement: Supplementary file 1 — Supplementary Materials [file 41389_2018_112_MOESM1_ESM.pdf]

Supplementary Information

Histone demethylase KDM6B has an anti-tumorigenic function in neuroblastoma by promoting differentiation

Liqun Yang, Yunhong Zha, Jane Ding, Bingwei Ye, Mengling Liu, Chunhong Yan, Zheng Dong, Hongjuan Cui, and Han-Fei Ding

Supplementary Figures 1-5
Supplementary Table S3-S4

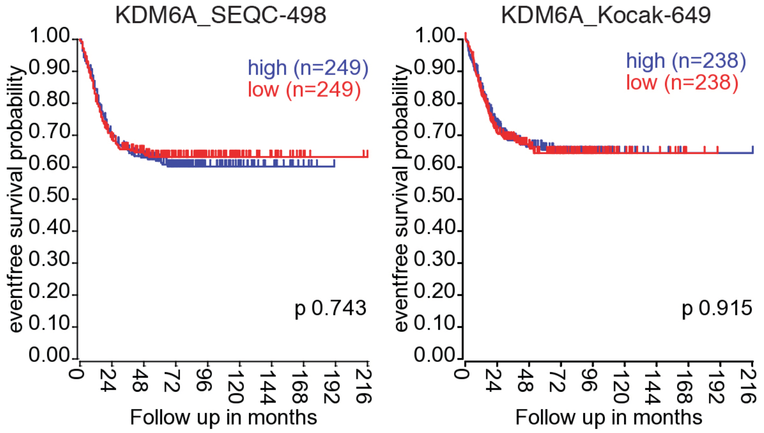

Supplementary Figure 1. *KDM6A* expression is not prognostic for the survival of neuroblastoma patients. Event-free Kaplan-Meier survival curves for the SEQC and Kocak cohorts of neuroblastoma patients based on *KDM6A* mRNA expression. Log-rank test *p* values are indicated.

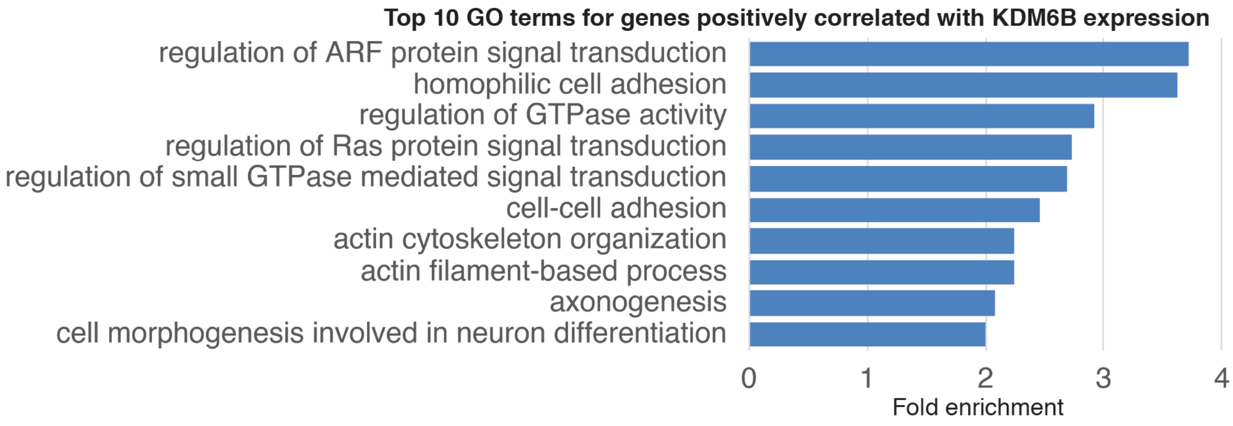

Supplementary Figure 2. High *KDM6B* mRNA expression is associated with neuronal differentiation of neuroblastoma tumors. Top GO terms for genes that are positively correlated with *KDM6B* in mRNA expression ($r \geq +0.25$, see also **Supplementary Table S1**) in neuroblastoma tumors from the SEQC cohort (n = 498). Gene sets were ranked based on fold enrichment with FDR < 0.01 (see also **Supplementary Table S2**).

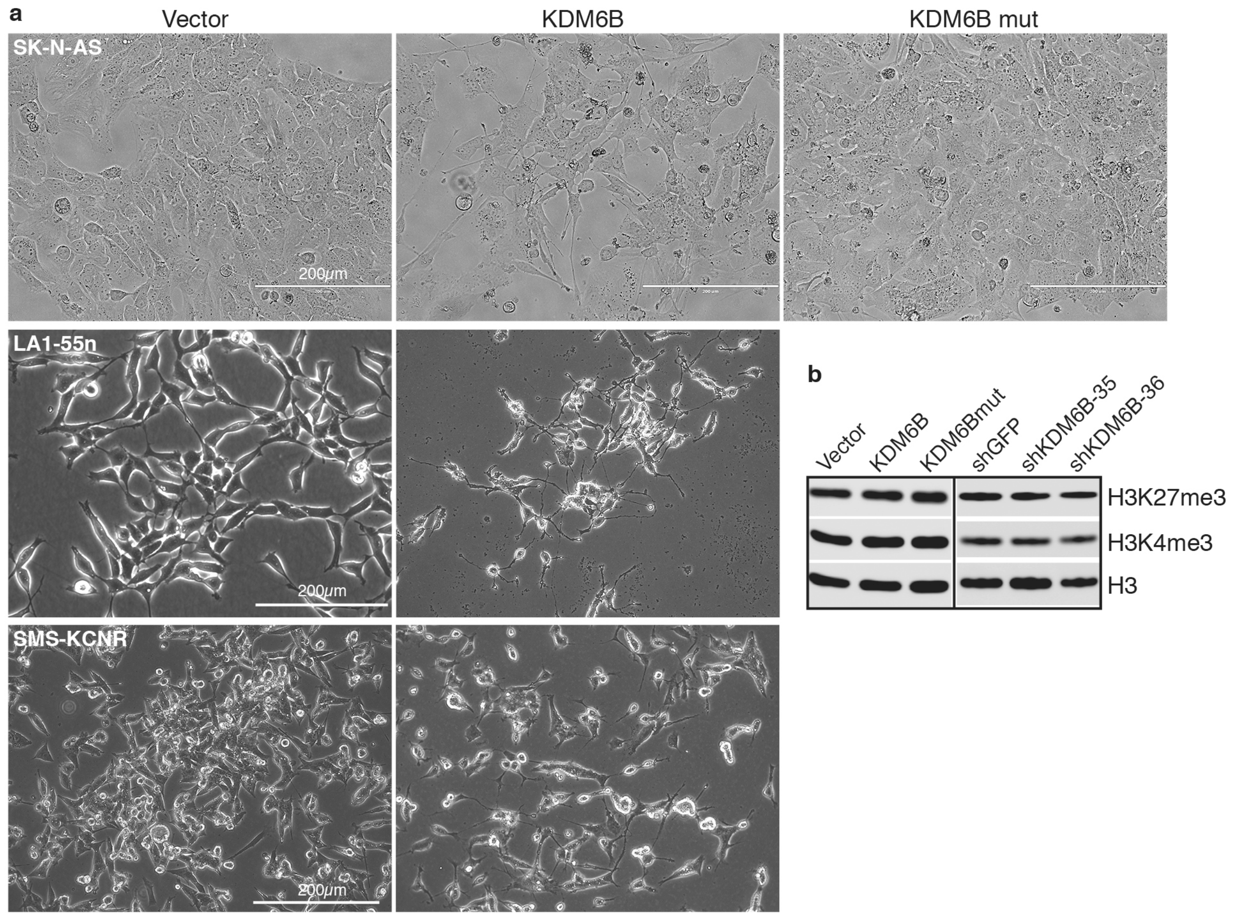

Supplementary Figure 3. KDM6B induces neuroblastoma cell differentiation. **a,** Phase contrast images of the indicated neuroblastoma cell lines without (vector control) or with overexpression of KDM6B or KDM6B-H1390A. **b,** Immunoblot analysis of H3K27me3, H3K4me3, and histone H3 levels in BE(2)-C cells with overexpression of KDM6B or its mutant or with KDM6B knockdown by shRNA. Vector and shGFP were used as control.

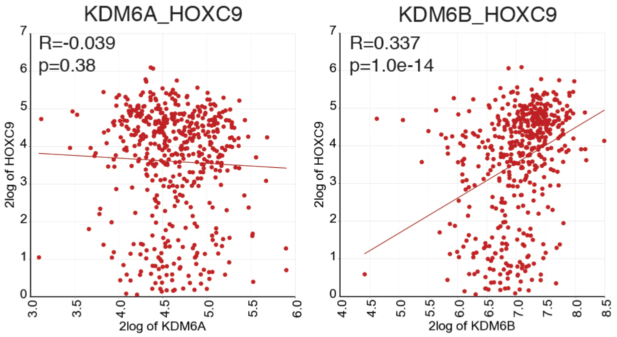

Supplementary Figure 4. *HOXC9* expression is correlated positively with *KDM6B* expression, but not with *KDM6A* expression, in primary neuroblastoma tumors (the SEQC dataset, n = 498). R (correlation) and *p* values are indicated.

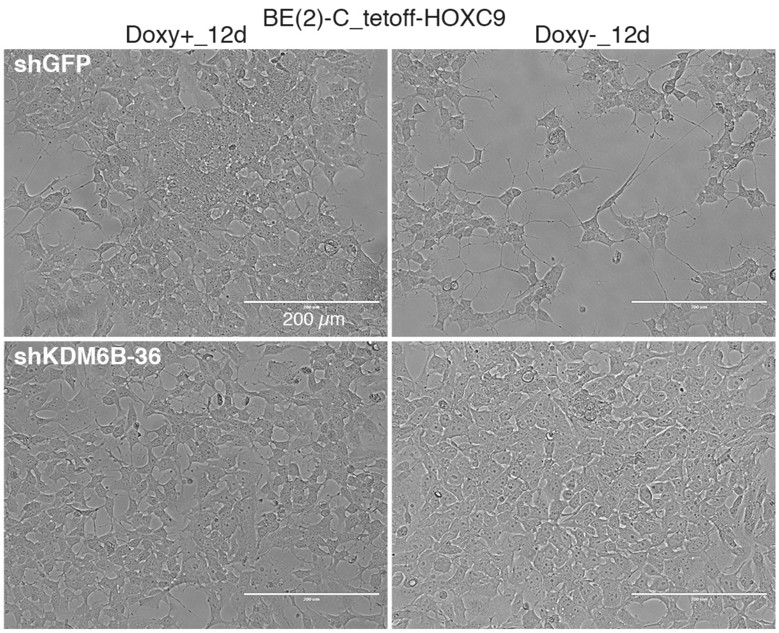

Supplementary Figure 5. KDM6B is required for HOXC9-induced neuronal differentiation. Phase contrast images of BE(2)-C_tetoff-myc-HOXC9 cells expressing either shGFP or shKDM6B-36 that were cultured in the presence or absence of Doxy for 12 days, showing that KDM6B knockdown abolished HOXC9-induced growth arrest and neurite outgrowth.

Supplementary Table S3. qRT-PCR primers

Primer set	Forward	Reverse
HOXC9	CTCGCTCATCTCTCACGACAA	GACGGAAAATCGCTACAGTCC
GFRA3	CCTACCTTTGCTGTGATGGCAC	CTACCATAGGCTCAGGAGCAGA
KDM6A	AGCGCAAAGGAGCCGTGGAAAA	GTCGTTCACCATTAGGACCTGC
KDM6B	GACCCTCGAAATCCCATCACAG	GTGCGAACTTCCACGGTGTGTT
NEFM	AGTGGTTCAAATGCCGCTAC	TTTTCCAACTGCTGGATGGT
RET	GGCATCAACGTCCAGTACAAG	TGAGGTGACCACCCCTAGC
B2M	TGCTGTCTCCATGTTTGATGTATCT	TCTCTGCTCCCCACCTCTAAGT

Supplementary Table S4. ChIP-qPCR primers

Primer set	Forward	Reverse
KDM6Bp_-1280	GTGGGAGCCAGTTGTGCTTGG	GACCCAGACACCATCCTGGGG
KDM6Bp_-469	AAGCCTTAAGTCTCTCAATGC	CTCCGCCCTGCCCATTCGTTC
KDM6Bp_+11281	TACACTGGCAGCTCTGGTTTT	AAGGGCAAAGGCTTCCCGTGC
KDM6Bp_+19291	CCAGCCAGCACGTCGCGATGA	TAATAAATTAAGTGAGGGGAG
NEFMp_-2k	CCTTTCCTGATTACTTACTGA	AGGGACTCCAGACCGAAATAG
NEFMp_-1k	TGCCAACCTGACAGGCCAGGC	GGCAGAAGGTAAGGCGCTTGT
NEFMp_+1	TAAATGGGCTGCGGCGAGGCC	TGCGAGCGGAAGCCACTGGAC
